# Spectroscopic Investigation of the Mechanism of Photocatalysis

**DOI:** 10.3390/molecules191118248

**Published:** 2014-11-07

**Authors:** Yoshio Nosaka, Masami Nishikawa, Atsuko Y. Nosaka

**Affiliations:** Nagaoka University of Technology, 1603-1 Kamitomioka, Nagaoka 940-2188, Japan; E-Mails: nishikawa@nagaokaut.ac.jp (M.N.); aynosaka@mst.nagaokaut.ac.jp (A.Y.N.)

**Keywords:** ESR, NMR, OH radical, superoxide radical, H_2_O_2_, acetaldehyde, metal doped TiO_2_, glutathione, amino acids, visible-light responsive photocatalysts

## Abstract

Reaction mechanisms of various kinds of photocatalysts have been reviewed based on the recent reports, in which various spectroscopic techniques including luminol chemiluminescence photometry, fluorescence probe method, electron spin resonance (ESR), and nuclear magnetic resonance (NMR) spectroscopy were applied. The reaction mechanisms elucidated for bare and modified TiO_2_ were described individually. The modified visible light responsive TiO_2_ photocatalysts, *i.e.*, Fe(III)-deposited metal-doped TiO_2_ and platinum complex-deposited TiO_2_, were studied by detecting paramagnetic species with ESR, •O_2_^−^ (or H_2_O_2_) with chemiluminescence photometry, and OH radicals with a fluorescence probe method. For bare TiO_2_, the difference in the oxidation mechanism for the different crystalline form was investigated by the fluorescence probe method, while the adsorption and decomposition behaviors of several amino acids and peptides were investigated by ^1^H-NMR spectroscopy.

## 1. Introduction

TiO_2_ photocatalysts have been widely utilized for the oxidation of organic pollutants [1,2,3,4]. For further practical applications, the improvement in the photocatalytic efficiency and the extension of the effective wavelength to visible region are desired. To develop photocatalysts, understanding of the detailed photocatalytic mechanisms is prerequisite. Recently, the reaction mechanisms of TiO_2_ photocatalysis have been extensively reviewed [5] and the authors also reviewed the reports published up to 2011 from the view of the detection of active oxygen species [6]. In this manuscript, recent development in the reaction mechanism mainly reported by our group was reviewed. Main techniques used were ESR spectroscopy for the state of photoinduced electron and holes, fluorescence probe method for the formation of OH radical and NMR spectroscopy for the adsorption and decomposition of biological molecules in solution.

## 2. Spectroscopic Methods for Investigating Photocatalysis

### 2.1. ESR (Electron Spin Resonance) Spectroscopy

ESR spectroscopy is conventionally used to detect unpaired electrons. Photocatalytic reactions proceed by the two following reactions: reduction of reactants with photoexcited electrons and oxidation of reactants with holes. Therefore, it is important to examine the generation behavior of these active species. In TiO_2_ photocatalytic systems, two kinds of active species (photoexcited electron and hole) are generated on absorbing photons. Some of the electrons and holes are trapped at Ti and O atoms, to become Ti^3+^ and O^−^, respectively. Therefore, by detecting these trapped electrons and holes using ESR spectroscopy under light irradiation of different wavelengths, the generation behavior of excited species can be examined. Moreover, the electron transfer between photocatalysts and co-catalysts can be also examined because if the electron transfer occurs, the amount of the unpaired electron in the co-catalyst should change before and after light irradiation. Therefore, ESR spectroscopy is very useful to elucidate photocatalytic reaction mechanism.

### 2.2. Chemiluminescence Photometry

Reduced oxygen molecules such as superoxide radical (•O_2_^−^) and H_2_O_2_ can be detected by chemiluminescence with luminol (LH_2_, aminodiazabenzoquinone). The one electron oxidized state of luminol (•L^−^) reacts with •O_2_^−^ to form the excited state of 3-aminophthalic acid to emit fluorescence in alkaline solution [7], where •L^−^ is formed from LH_2_ by the oxidation with •O_2_^−^ [8]. Since •O_2_^−^ is rather stable in alkaline solution, after the irradiation on photocatalyst was stopped, luminol is injected to measure the amount of •O_2_^−^ by the chemiluminescence intensity. The same chemiluminescence was obtained from H_2_O_2_ by the reaction with L that is two-electron oxidized state of LH_2_ [9]. To measure the amount of H_2_O_2_ in solution, after mixing luminol, hemoglobin was added to oxidize luminol, because L is rather unstable [8]. Luminol chemiluminescence method has some problems. It is available only in alkaline solution, and luminol emits light with SiO_2_ in the absence of •O_2_^−^ and H_2_O_2_. Therefore, in the case of SiO_2_ deposited TiO_2_, instead of luminol, MCLA and lucigenin were employed for the detection of •O_2_^−^ and H_2_O_2_, respectively, by means of chemiluminescence photometry [10].

### 2.3. Florescence Probe Method

**Figure 1:**
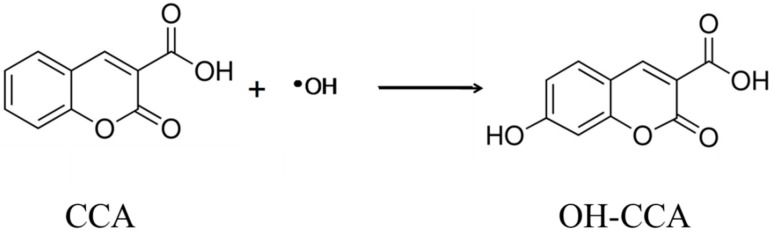
Probing reaction of OH radical with CCA (coumarin 3-carboxy acid) to form fluorescent molecule OH-CCA (7-hydroxy coumarin 3-carboxy acid).

Hydroxyl radical (•OH) has been recognized as a key active species in the oxidation mechanism in photocatalysis [9,11]. For the detection of •OH we employed coumarin. It reacts with •OH to produce 7-OH coumarin (umbelliferone) which emits strong fluorescence [12]. After the irradiation of a coumarin aqueous solution containing photocatalysts powder for a given time, the fluorescence intensity of the fluorescent products (umbelliferone) in the solution was measured. The •OH concentration could be calculated from the concentration of umbelliferone with the aid of data of radiation chemistry [12]. Since carboxyl group is known to adsorb on TiO_2_, the similar experiments were performed for 3-carboxylic acid derivative of coumarin (CCA, Figure 1), and ensured the reaction with •OH to form OH-CCA as illustrated in Figure 1 [13].

### 2.4. NMR (Nuclear Magnetic Resonance) Spectroscopy

^1^H-NMR spectroscopy has been recognized as an effective technique to investigate the behaviors of the reactant molecules in the photocatalytic systems. The adsorption and the decomposition of biomolecules such as amino acids and peptides in the aqueous suspension of photocatalysts can be investigated with ^1^H-NMR spectroscopy with relatively feasible experimental procedures [14] as follows. Firstly, ^1^H-NMR of organic molecules dissolved in the solvent are measured. Then certain amount of the photocatalysts is added to the solution. From the initial decrease in the intensity of ^1^H-NMR peaks of the corresponding reactant molecules the amount of the adsorption can be estimated. Then, by measuring the decrease in the intensities of reactant molecules for various photoirradiation times, one could estimate the photodecomposition rates of the reactants [15].

## 3. Mechanism of Photocatalysis

### 3.1. Bare-TiO_2_ and Visible-Light Responsive TiO_2_ Photocatalysts

**Figure 2:**
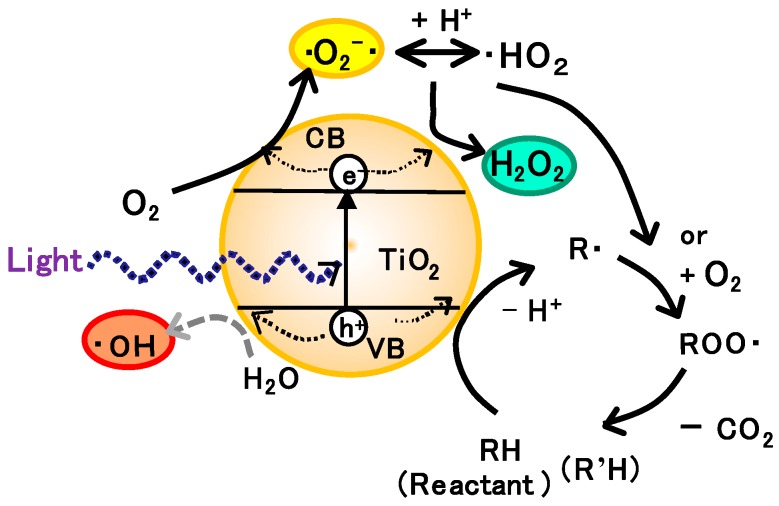
General reaction processes for the photocatalytic oxidation of organic molecules.

General scheme of photocatalysis applied for the oxidation of pollutant is shown in Figure 2. Light absorption in semiconductor corresponds to the formation of an electron (e^−^) in the conduction band (CB) and a hole (h^+^) in the valence band (VB). Usually e^−^ reduces O_2_ in air to form •O_2_^−^ and H_2_O_2_.

The photocatalytic oxidation of organic compounds is accelerated with oxygen [16]. The consumption of O_2_ at the oxidation site of the photocatalyst has been suggested from the experiment of electrochemical probe reactions at the surface of illuminated TiO_2_ photoelectrode [17]. Therefore, the generalized oxidation mechanism of organic molecules (RH) can be illustrated as shown in Figure 2. Organic reactants RH will degrade by losing one carbon atom by releasing CO_2_ through the intermediates like aldehyde R’CHO or carboxylate R’COO^−^. Although •OH has been often regarded to play an important role in the actual oxidation mechanism of photocatalytic reactions, •OH is not involved in the main oxidation process for organic compounds. In place of it, the surface trapped holes play the role of oxidation, which may be acknowledged as the surface adsorbed •OH in the de-protonated form as stated below.

**Figure 3:**
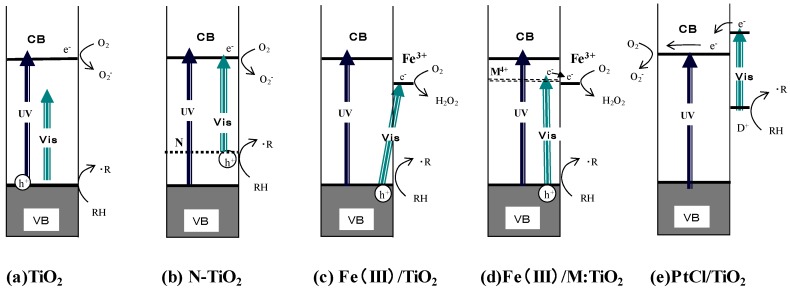
Classification of visible-responsive photocatalysts by the primary reaction mechanism. (**a**) Unmodified TiO_2_; (**b**) Nitrogen doped TiO_2_; (**c**) Fe(III) grafted TiO_2_; (**d**) Fe(III) grafted metal doped TiO_2_; (**e**) Platinum complex deposited TiO_2_. Adopted with permission from [18]. © 2013 American Chemical Society.

For the extension of the practical applications of photocatalysts, the utilization of visible light has been intensively promoted. Figure 3 shows the energy levels of several representative visible-light responsive photocatalysts. Since the one-electron reduction potential of O_2_ is very close to that of the CB bottom of TiO_2_ and the energy level of VB has sufficient oxidation ability, the shift of the VB by doping N (or, C and S) anions has been attempted to absorb visible light (b). In this case, photogenerated holes at the donor level should have the oxidation ability similarly to that of bare TiO_2_ [9].

Recently, interfacial charge transfer (IFCT) type absorption originating from the excitation of VB electrons to deposited (or grafted) metal ions has been proposed (c). In this case, if the deposited compound has a catalytic ability of O_2_ reduction, the efficiency is expected to be increased [19,20]. Since the absorbance of IFCT is very small, to increase the absorption, the transferring of the excited electron to the graft level by doping of metal ions was proposed (d). Photocatalysts of photosensitization type were also proposed, in which the stable metal complex such as PtCl_6_^2^^−^ is deposited as a sensitizer (e). The deposited compound absorbs the visible light and transfers the excited electron to produce a cation radical D^+^, which can oxidize organic pollutant molecules. In this case, the enough oxidation power with good stability is required for the oxidized sensitizer D^+^ [21,22]. To confirm the suggested reaction mechanism, several spectroscopic methods have been applied to the detection of the paramagnetic species produced on the catalysts along with the primary products (•O_2_^−^, H_2_O_2_, •OH).

#### 3.1.1. Fe(III) Grafted TiO_2_ Based Photocatalysts

**Figure 4:**
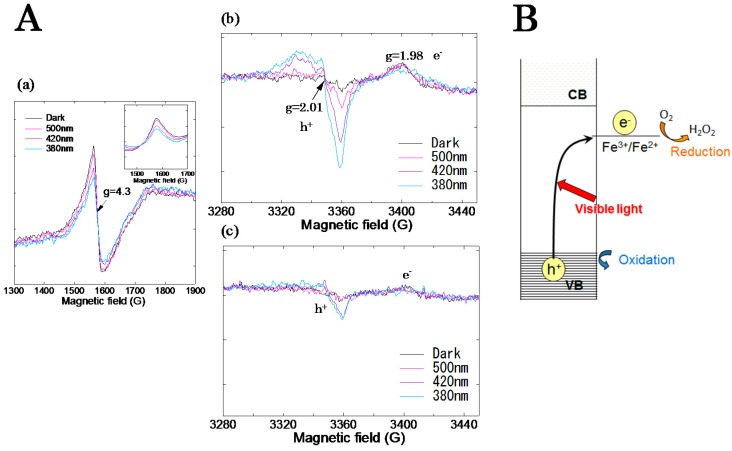
(**A**) ESR spectra of (**a**) Fe^3+^ and (**b**) holes for Fe/TiO_2_ and (**c**) holes for TiO_2_ before and after light irradiation with different wavelengths; (**B**) Photocatalytic reaction mechanism for Fe/TiO_2_ under visible light irradiation. Reprinted with permission from [23]. © 2012 American Chemical Society.

Fe(III) grafted TiO_2_ (Fe/TiO_2_) showed the photocatalytic activity under visible light irradiation. The quantum efficiency of Fe/TiO_2_ prepared under optimized condition was reported to be 22% [20]. We examined the photocatalytic reaction mechanism of the Fe/TiO_2_ using ESR spectroscopy [23]. As shown in Figure 4A, under visible light irradiation, the ESR signal assigned to Fe^3+^ (g = 4.3) was decreased and the ESR signal assigned to trapped holes (g = 2.01) at the TiO_2_ host was observed. In the case of TiO_2_ without the grafting of Fe^3+^, the trapped hole signal was scarcely observed as compared to the Fe/TiO_2_ under visible light irradiation. This means that electrons at VB are directly transferred to the grafted Fe^3+^ rather than CB (Figure 4B). Using ESR spectroscopy, we could reveal for the first time that the direct electron transfer from the VB of TiO_2_ to the Fe^3+^ is the origin of the visible light response.

Photocatalytic reactions cannot proceed when the photogenerated electrons do not react with molecular oxygen, which is the only molecule to be reduced in ambient atmosphere even though the redox potential of the photogenerated hole is positive enough to decompose organic compounds. Therefore, it is important to confirm the reduction of O_2_ into •O_2_^−^ by one-electron or H_2_O_2_ by two-electron reductions. Under visible light irradiation, for Fe/TiO_2_, the production of H_2_O_2_ was dominant as compared to •O_2_^−^. This means that the excited Fe^2+^ can reduce O_2_ to H_2_O_2_ through two-electron process. Since the electrons having a potential energy of +0.695 V (*vs.* SHE at pH = 0) can reduce O_2_ to H_2_O_2_ by two-electron process [24], the redox potential of the grafted Fe^3+^ to Fe^2+^ was equal to or less than +0.695 V (*vs.* SHE at pH = 0). Since photogenerated electrons were consumed by the reduction of O_2_ to H_2_O_2_, holes remained at valence band could decompose organic substances efficiently, resulting in the high performance. 

**Figure 5:**
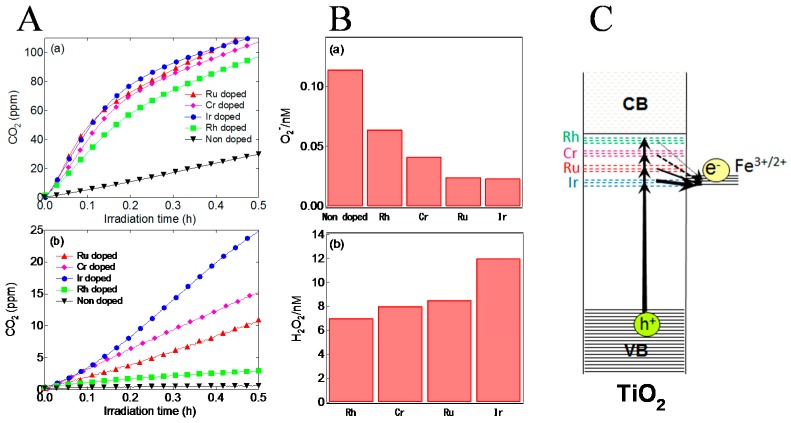
(**A**) Photocatalytic activities of the Fe/M:TiO_2_ against gaseous acetaldehyde under visible light irradiation of (**a**) λ = 470 nm and (**b**) λ = 625 nm; (**B**) The amount of (**a**) •O_2_^−^ and (**b**) H_2_O_2_ generated on the M:TiO_2_ and the Fe/M:TiO_2_, respectively under visible light irradiation of λ = 625 nm; (**C**) Schematic energy level diagram for the Fe/M:TiO_2_. Reprinted with permission from [25]. © 2014 Elsevier B. V.

Moreover, after the grafting of Fe^3+^ on the TiO_2_ doped with metal (M) ions such as Ru, Ir, Rh or Cr ions (Fe/M:TiO_2_), the photocatalytic activities were enhanced compared to the Fe/TiO_2_ as shown in Figure 5A. The visible light response was increased in the order Ir > Cr > Ru > Rh. In the case of the Fe/Ru:TiO_2_, based on the measurements by ESR spectroscopy, Ru ions were doped as tetravalent and played a role as an acceptor level. Then, the photoexcited Ru ions, by receiving electrons from the VB, immediately transfer electrons to the grafted Fe^3+^ under visible light irradiation. This indirect electron transfer from the VB to the Fe^3+^ via the doped Ru ions occurred in addition to the direct electron transfer from the VB to the Fe^3+^, leading to the enhancement of photocatalytic activity.

Furthermore, we examined the desirable character of doped metal ions for photocatalytic performance in detail. Figure 5B shows the amounts of •O_2_^−^ and H_2_O_2_ generated on the M:TiO_2_ and the Fe/M:TiO_2_, respectively, under visible light irradiation. The electrons having a potential energy of +0.38 V (*vs.* SHE at pH = 0) can reduce O_2_ into •O_2_^−^ under the experimental conditions (pH = 11.5) [24]. Therefore, by measuring the amount of •O_2_^−^, the redox potential of dopants can be relatively estimated. For the non-doped TiO_2_, the generation amount of •O_2_^−^ was larger than that of the M:TiO_2_. For the non-doped TiO_2_, O_2_ was reduced to •O_2_^−^ by an electron excited at conduction band from defect level under visible light irradiation. This result indicated that all kinds of dopant used played a role as acceptor, because if they play a role as donor, the generation amount of •O_2_^−^ should increase due to electron excitations from dopants to CB. Among the M:TiO_2_, the generation amount of •O_2_^−^ was decreased in the order of Rh > Cr > Ru > Ir. Since the order of the redox level of dopants should be consistent with that of the •O_2_^−^ amount, their redox levels would be more negative in the order of Rh > Cr > Ru > Ir. Secondly, when electrons transfer to the grafted Fe^3+^, the excited Fe^2+^ can reduce O_2_ to H_2_O_2_ by a two-electron process as mentioned below. Therefore, we can determine the degree of electron transfer to the Fe^3+^ from the dopants by the measurement of amount of H_2_O_2_. The generation amount of H_2_O_2_ was decreased in the order of Ir > Ru > Cr > Rh (Figure 5B). This H_2_O_2_ generation tendency was opposite to that of •O_2_^−^. This means that through the dopant with more positive redox potential, electrons can transfer more easily to the Fe^3+^ as illustrated in Figure 5C. This is due to the small energy loss of electrons when the redox potential of dopant was close to that of the Fe^3+^. Therefore, we concluded that the high photocatalytic activity of the Fe/Ir:TiO_2_ under visible light irradiation was attributable to the acceptor level due to Ir^4+^ formed close to the redox potential of the grafted Fe^3+^ (Figure 5C).

**Figure 6:**
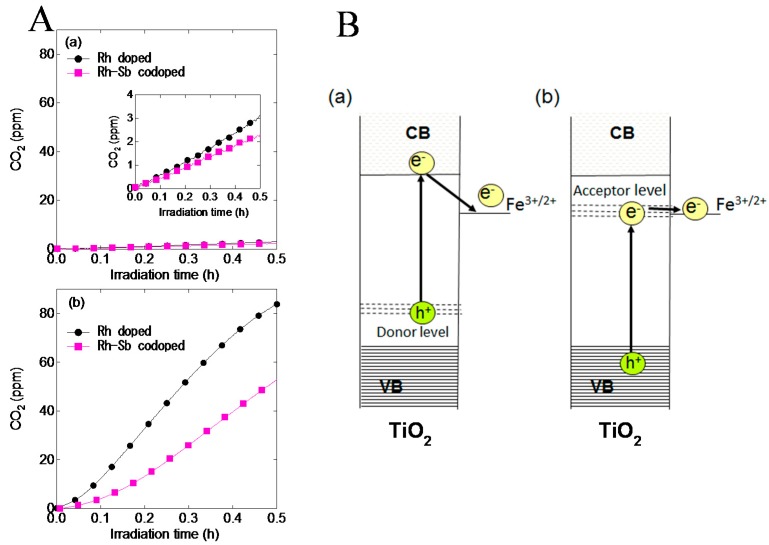
(**A**) The co-doped effect on photocatalytic activities for CO_2_ formation from gaseous acetaldehyde under visible light irradiation of λ = 470 nm. (**a**) Before and (**b**) after grafting of Fe^3+^; (**B**) Indirect electron transfer paths to the Fe^3+^ via (**a**) donor level and (**b**) acceptor level. Reprinted with permission from [25]. © 2014 Elsevier B. V.

In the case of TiO_2_ codoped with Rh and Sb ions, the efficiency of the indirect electron transfer to the Fe^3+^ was lowered compared to the TiO_2_ doped with Rh ions alone (Figure 6A). By codoping with Sb ions, Rh^4+^ was reduced to Rh^3+^ and the formed Rh^3+^ played a role as donor [26,27]. This indicated that the efficiency of the indirect electron transfer of Rh^3+^ → CB → Fe^3+^ was lower than that of VB → Rh^4+^ → Fe^3+^ (Figure 6B).

The energy gap of the redox potential between conduction band and the grafted Fe^3+^ was larger than that between the doped Rh^4+^ and the grafted Fe^3+^ and therefore electrons photoexcited at CB could not effectively transfer to the Fe^3+^ because of the large energy loss. From these results, forming acceptor level closed to the redox potential of the grafted Fe^3+^ was important for high performance of Fe/TiO_2_ based photocatalysts under visible light irradiation [25]. Recently, a better energy level matching in Fe/M:TiO_2_ was achieved by employing Fe^3+^ as a doping metal ion [28].

#### 3.1.2. Pt Chloride Deposited TiO_2_ Photocatalysts

Pt^4+^ chloride deposited TiO_2_ (PtCl/TiO_2_) also showed a photocatalytic activity under visible light irradiation and its quantum efficiency was 9.8% [20]. In the past, Kisch *et al.*, reported a mechanistic hypothesis to explain PtCl/TiO_2_ activity [21,29]. The proposed hypothesis was that the PtCl undergoes homolytic Pt-Cl cleavage by absorbing of light, generating a Pt^3+^ intermediate and a chlorine atom, the Pt^3+^ injects an electron to the conduction band of TiO_2_, and then the Cl radical oxidizes organic compounds. However, it is not clear whether the Pt-Cl cleavage in the PtCl/TiO_2_ system would occur. In addition, there is no sufficient evidence to support the injection of electron from Pt^3+^ to the conduction band of TiO_2_. Therefore, we clarified the charge transfer between the PtCl and TiO_2_ under visible-light irradiation using ESR spectroscopy [30].

**Figure 7:**
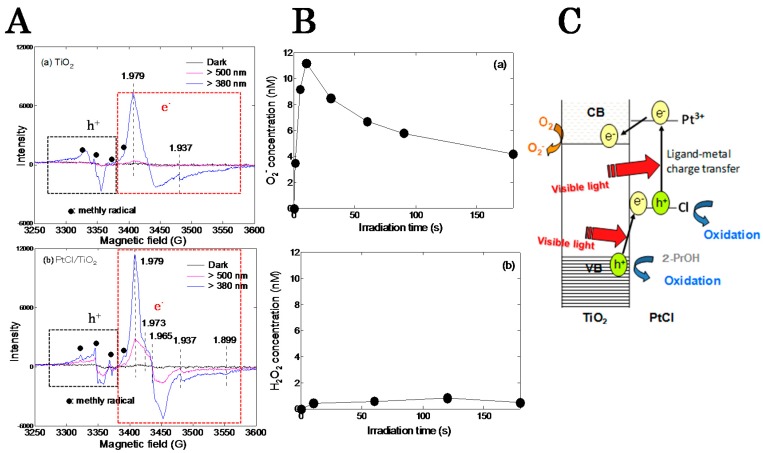
(**A**) ESR spectra of electrons and holes for (**a**) TiO_2_ and (**b**) PtCl/TiO_2_; (**B**) (**a**) •O_2_^−^ and (**b**) H_2_O_2_ generated on the PtCl/TiO_2_ under visible light irradiation. (**C**) Photocatalytic reaction mechanism of PtCl/TiO_2_. Reprinted with permission from [30]. © 2012 American Chemical Society.

For a bare TiO_2_ without deposition of PtCl, under visible light irradiation, both ESR signals assigned to trapped electrons and holes were not observed (Figure 7A(a)). For the PtCl/TiO_2_, a signal assigned to Pt^3+^ was observed. This means that Pt^4+^ chloride complexes were charge-separated into Pt^3+^ and Cl radicals. Then in the TiO_2_ host, trapped electrons (g ≈ 1.98) were observed (Figure 7A(b)). These results proved that TiO_2_ could receive electrons from excited Pt^3+^ as well as the hypothetical mechanism. However, unlike the hypothetical mechanism, trapped hole signal (g = 2.01) was also observed. Based on the results, some electrons in the VB of TiO_2_ would be excited to the orbital of the Cl radicals similarly to the case of direct electron transfer from the VB of TiO_2_ to the grafted Fe^3+^ for the Fe/TiO_2_ photocatalysts. Since the redox potential (+3.0 V *vs.* SHE at pH = 0) of the VB of rutile TiO_2_ is more positive than that (2.47 V *vs.* SHE at pH = 0) of Cl/Cl^−^ [31], the high photocatalytic activity of PtCl/TiO_2_ would be owing to the generation of holes in the TiO_2_ host.

Generation behaviors of •O_2_^−^ and H_2_O_2_ under visible light irradiation were also examined for the PtCl/TiO_2_ as shown in Figure 6B. •O_2_^−^ was predominantly generated compared to H_2_O_2_. This was opposite behavior to the Fe/TiO_2_ for which H_2_O_2_ was dominantly generated rather than •O_2_^−^. This means that photoexcited electrons have a higher potential energy than +0.38 V (*vs.* SHE at pH = 0) and therefore the reduction of O_2_ to •O_2_^−^ was produced by the electrons photoexcited at CB of TiO_2_, which supported the ESR results [30].

A plausible reaction mechanism for the PtCl/TiO_2_ photocatalyst is illustrated in Figure 7C. Photoexcited Pt^3+^ generated by ligand- metal charge transfer in deposited PtCl complex by adsorption of visible light gives an electron to the TiO_2_ CB and then the electron is consumed by reduction of O_2_ into •O_2_^−^. Some of the photogenerated Cl radicals can decompose organic substances and the other receive electrons by photo-excitation from the valence band of TiO_2_, resulting the generation of holes in TiO_2_. The organic substances can be efficiently decomposed by the generated holes in TiO_2_ with strong oxidation ability.

#### 3.1.3. Comparison of the Visible-Light Responsive TiO_2_ Photocatalysts

**Figure 8:**
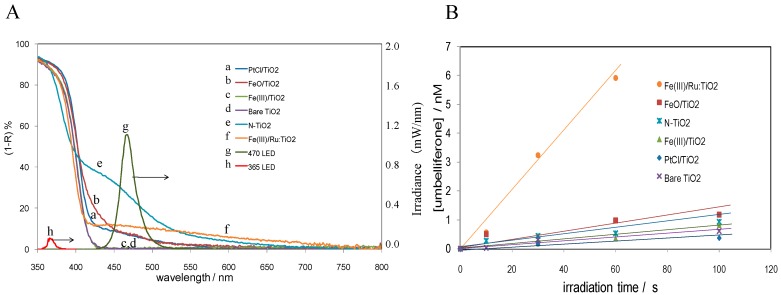
(**A**) Absorption spectra as the complement of the reflectance (1-R) and the irradiance spectra of LED used in the study; (**B**) Concentration of umbelliferone generated under 470-nm irradiation in aqueous coumarin solution was plotted as a function of the irradiation time. Reprinted with permission from [18]. © 2013 American Chemical Society.

The reaction mechanisms of various modified TiO_2_ were investigated by detecting •OH quantitatively by means of a coumarin fluorescence probe method [18]. The photocatalysts investigated were nitrogen-doped, Fe(III)-grafted, Fe(III)-grafted Ru-dopedTiO_2_, and Pt-complex-deposited, whose diffuse reflectance spectra are shown in Figure 8A. On the irradiation with 470 nm light in the presence of coumarin, the concentration of umbelliferone was increased (Figure 8B). From the slope, the formation rate of •OH was calculated. Then, the •OH quantum yield was calculated with the absorbed light intensity which was evaluated from the absorption and irradiance spectra in Figure 8A. The quantum yield ranged from 10^−5^ for N-TiO_2_ to 4 × 10^−4^ for Fe/TiO_2_ [18]. In the presence of 0.14 mM H_2_O_2_, the •OH yield decreased for N-TiO_2_ while it increased for Fe/TiO_2_. The increase for Fe/TiO_2_ suggests that H_2_O_2_ is a reaction intermediate for producing •OH.

**Figure 9:**
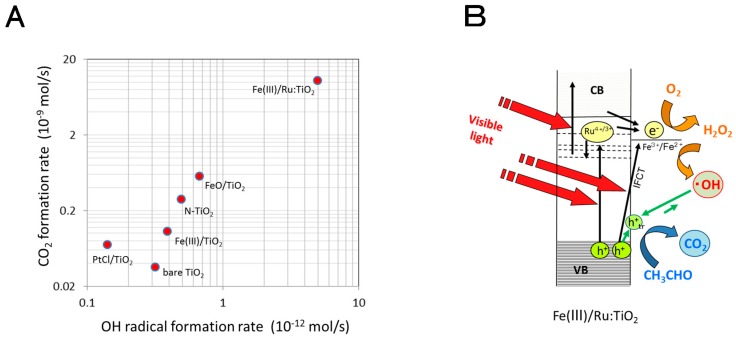
(**A**) Relationship between the formation rates of CO_2_ and •OH under the irradiation of 470 nm LED. The CO_2_ formation rate is a measure of the photocatalytic reaction rate in the acetaldehyde decomposition in aqueous suspension system; (**B**) Schematic illustration of reaction mechanism of Fe(III)-grafted Ru-doped TiO_2_ photocatalyst based on the detection of •OH and CO_2_. Key: IFCT, interfacial charge transfer; h^+^_tr_, surface trapped hole. Reprinted with permission from [18]. © 2013 American Chemical Society.

The photocatalytic activity was evaluated by the rate of CO_2_ generation associated with acetaldehyde decomposition and then it was plotted in Figure 9A as a function of the •OH formation rate for each photocatalyst. The CO_2_ generation rates of the photocatalysts were positively correlated with those of the •OH formation. However, the formation rates of CO_2_ were extremely larger (10^3^ times) than those of •OH. This finding indicates that the oxidation reaction predominantly takes place at the photocatalyst surface with the trapped holes. The good correlation in the figure suggests that •OH in the bulk solution is equilibrated with trapped holes (Equation (1)), but the equilibrium is significantly shifted to the surface trapped holes.

•OH + Ti^4+^[TiO_2_] ←→ •O^−^Ti^4+^[TiO_2_] + H^+^ (1)

The highest photocatalytic activity in the suspension system was obtained for Fe(III)-deposited Ru-doped TiO_2_ (Fe(III)/Ru:TiO_2_) whose reaction mechanism is shown in Figure 9B. On the basis of the ESR and chemiluminescence experiments mentioned above [23], the CB electrons are formed by two step excitation with visible light irradiation and O_2_ is reduced to H_2_O_2_. The grafted Fe^3+^ is reduced by Ru^3+^ or by IFCT and then the formed Fe^2+^ produces •OH from H_2_O_2_. The •OH produced in solution is adsorbed on the TiO_2_ surface to form trapped holes which could oxidize organic compounds, such as acetaldehyde, leading to CO_2_.

### 3.2. Photocatalysis with Bare TiO_2_

#### 3.2.1. Reactivities of Rutile and Anatase Surfaces

**Figure 10:**
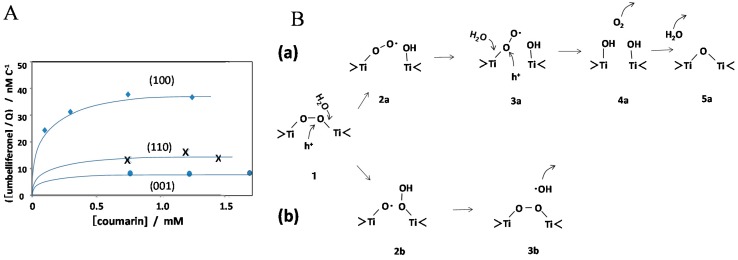
(**A**) The amount of produced umbelliferone normalized for the current charge is plotted against the coumarin concentration in solution for the rutile (100), (110), and (001) TiO_2_ electrodes; (**B**) Plausible reaction steps starting from peroxo to form (**a**) O_2_ and (**b**) •OH at the TiO_2_ surface. Reprinted with permission from [33]. © 2013 American Chemical Society.

The photogeneration of molecular oxygen at rutile TiO_2_ electrode is a famous historical reaction [32]. To investigate the oxidation mechanism, •OH formation was measured by employing three electrodes of rutile TiO_2_ (100), (110), and (001) [33]. Figure 10A shows the amount of the produced umbelliferone, which is normalized to the number of charges used in the reaction. For all electrodes, the photocurrent efficiency of •OH was less than 1%, while that of O_2_ was about 100%. This observation implies that the conventionally proposed mechanism to produce O_2_ via •OH formation is not a major mechanism in water oxidation at TiO_2_ surface. Figure 10B shows the plausible reaction steps in the formation of O_2_ and •OH through surface peroxo (Ti-O-O-Ti). By cleaving Ti-O bond in the peroxo, O_2_ is formed as shown in Figure 10B(a) [34]. When O-O bond is cleaved instead of Ti-O bond, •OH is formed as a byproduct. The •OH formation in Figure 10A increases in the order of (001) < (110) < (100), which can be explained by the strength of Ti-O bond deduced from the surface structure [33].

**Figure 11:**
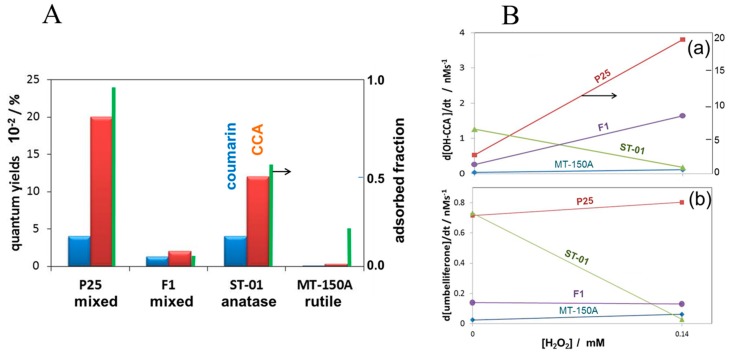
(**A**) Quantum yields of •OH using the different probe molecules, coumarin (blue) and CCA (brown), and the fraction of adsorbed CCA (green) for four kinds of TiO_2_ powders; (**B**) Effect of the addition of 0.14 mM H_2_O_2_ on the formation rates of (**a**) OH-CCA and (**b**) umbelliferone. Reprinted with permission from [36]. © 2014 American Chemical Society.

Though rutile TiO_2_ shows high activity for O_2_ evolution, anatase TiO_2_ is known to have a higher activity in the photocatalytic oxidation of organic molecules [35]. The difference in the generation process of •OH between rutile and anatase was investigated by using CCA and coumarin [36]. Figure 11A shows the quantum yields of •OH generation measured with coumarin and CCA together with the adsorbed fraction of CCA. As shown in Figure 11A, anatase and anatase-contained TiO_2_ (ST-01, P25, and F1) generated •OH in the substantial yields. The quantum yield for OH-CCA was much larger than that for umbelliferone, indicating that •OH is formed at the TiO_2_ surface and diffused into bulk solution. Furthermore, this observation indicates that the active site is different from the adsorption site of –COO^−^ group. Since H_2_O_2_ is produced in photocatalysis, the effect of H_2_O_2_ on the •OH generation was investigated. Figure 11B shows the effect of the addition of H_2_O_2_ on the formation rate for (a) OH-CCA and (b) umbelliferone. On the addition of H_2_O_2_, the •OH generation for pure anatase TiO_2_ decreased but increased for rutile and rutile-contained TiO_2_. This phenomenon has been reported previously for other several TiO_2_ powders [37]. The amount of •O_2_^−^ was significantly increased with the addition of H_2_O_2_ [37]. Although the formation of •OH from H_2_O_2_ by CB electrons is commonly suggested in the •OH generation mechanism, the fact that the •O_2_^−^ was significantly increased with H_2_O_2_ denied the one-electron reduction of H_2_O_2_.

**Figure 12:**
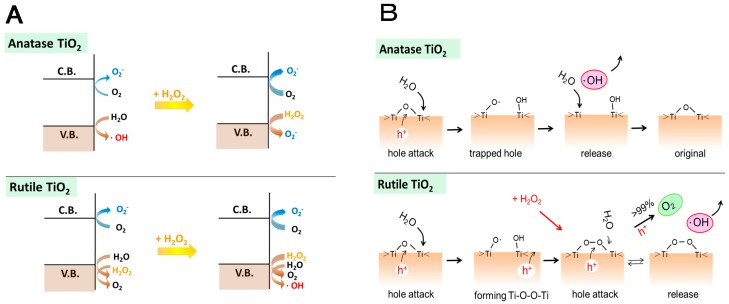
(**A**) Photocatalytic processes at the conduction band (C.B.) and the valence band (V.B.) of TiO_2_ with anatase and rutile crystalline types in the absence and the presence of H_2_O_2_. The thickness of arrows expresses the degree of the reaction rate; (**B**) Plausible mechanisms of •OH generation at anatase TiO_2_ (upper part) and rutile TiO_2_ (lower part). Reprinted with permission from [36]. © 2014 American Chemical Society.

Since the increase is remarkable for anatase than rutile TiO_2_, with the addition of H_2_O_2_, the generation of •OH at anatase surface was replaced by the oxidation of H_2_O_2_ to form •O_2_^−^, as illustrated in Figure 12A.

For rutile TiO_2_ adsorbed H_2_O_2_ is equivalent to the surface peroxo, Ti-O-O-Ti and promotes the formation of •OH as stated above. The detailed generation mechanism of •OH on anatase and rutile TiO_2_ surfaces can be proposed as shown in Figure 12B. On the anatase surface, photogenerated valence band holes, h^+^, are trapped at the surface oxygen to form trapped holes (Ti−O•) that can be regarded as the adsorbed •OH in the deprotonated form (•O^−^) [18] then an •OH is released to the solution as represented by Equation (1). On the other hand, for rutile TiO_2_, since the crystalline structure is packed more tightly than that for anatase, the stability of the surface trapped holes may be different. By trapping of h^+^ predominantly near the trapped hole, Ti-peroxo is formed. As described above in Figure 10B(b), •OH radical is produced by h^+^ from H_2_O with Ti-peroxo, which plays the role of a catalyst. Thus, the increase of the •OH generation with H_2_O_2_ for rutile TiO_2_ can be explained.

#### 3.2.2. Adsorption and Decomposition of Glycine Related Peptides

The application of photocatalysts to biological fields for their antibacterial effect and in medical treatments for diseases, including cancer, has been proceeding extensively [38,39]. It is believed that the active oxygen species generated on the photocatalysts such as H_2_O_2_, •OH, and singlet oxygen are involved in the attack to kill various kinds of virus and bacteria [40]. However, the mechanism underlying the photobiological activity is not yet well understood. Since the photocatalytic process is expected to occur at the interface between the photocatalysts and the liquid medium, the interface between protein molecules and inorganic materials has recently received much attention.

Proteins and peptides are composed of various kinds of amino acids. For a proper understanding of the adsorptive and photocatalytic interactions between the surface of the photocatalysts and proteins/peptides, fundamental knowledge on the adsorption and photocatalytic reactivity of individual constituent amino acids would be necessary.

TiO_2_ is widely used for practical applications as a photocatalyst. The surface of TiO_2_ is amphiphilic, which consists of hydrophobic and hydrophilic parts [41]. The hydrophilic parts involve two kinds of hydroxyl group, that is, the acidic bridged hydroxyl group and the basic terminal hydroxyl group. Both groups can be adsorptive and/or photocatalytic active sites, depending on the kinds of titanium dioxides which are characterized by different particle size, surface area, and crystal forms such as anatase, rutile and brookite. The photocatalyst with different characteristic surface shows different adsorbability and photocatalytic activity [40].

**Figure 13:**
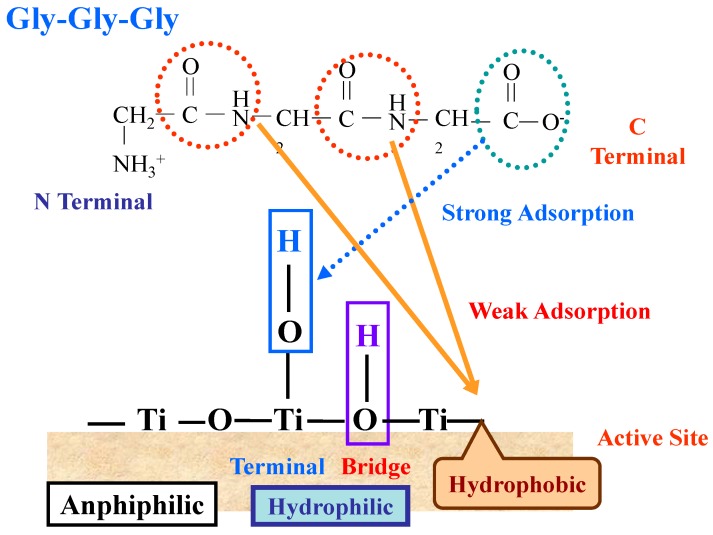
Schematic presentation of the adsorption of the peptide (Gly-Gly-Gly) on the hydrophilic and hydrophobic surface of TiO_2_ (ST-01). Reprinted with permission from [43] © 2014 American Chemical Society.

It was demonstrated that both hydrophilic and hydrophobic sites are adsorptive sites but that only hydrophobic sites are photocatalytically active for ST-01 TiO_2_ (100% anatase crystal form with a BET surface area of 320 m^2^·g^−1^ and a particle size of 9 nm; Ishihara Sangyo Ltd., Osaka, Japan) [15]. After the calcinations at 973 K hydrophilic parts of the surface of ST-01 can be eliminated and a highly hydrophobic surface (designated as HT-TiO_2_) is created without changing the crystal form [15]. By employing these characteristics, the adsorption and decomposition sites of the simplest amino acid glycine, whose adsorbability on the TiO_2_ surface is still controversial [42], and its homopeptides (Gly-Gly and Gly-Gly-Gly) were investigated by ^1^H-NMR spectroscopy [43]. For Gly-Gly and Gly-Gly-Gly the carboxylic group and the peptide bond were assigned as the adsorptive sites of the peptides on the surface of ST-01. The adsorption feature of Gly-Gly-Gly on TiO_2_ (ST-01) are illustrated in Figure 13; the peptide would adsorb by the *C*-terminal carboxyl group most probably with the terminal hydroxyl group at 5-coordinated Ti of TiO_2_ as is generally believed [42]. The photo decomposition took place by the weak adsorption of the peptide bonds on the surface of TiO_2_ (ST-01).

**Figure 14:**
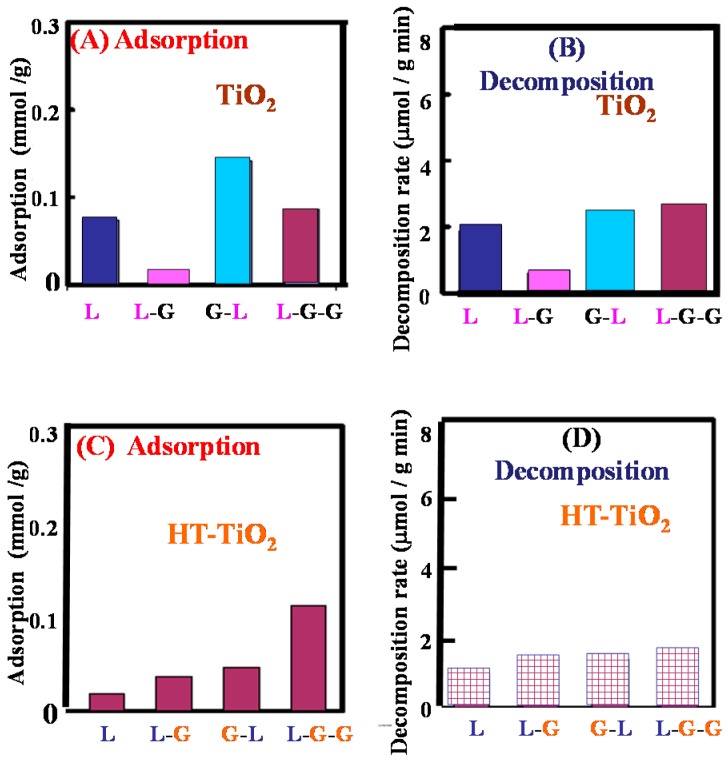
Equilibrium adsorption and rate of decomposition under the UV irradiation measured at 297 K for Leu, Leu-Gly, Gly-Leu, and Leu-Gly-Gly in the aqueous suspensions of TiO_2_; (**A**,**B**) for untreated TiO_2_ and (**C**,**D**) for HT-TiO_2_ (TiO_2_ calcined at 973 K). Reprinted with permission from [43] © 2014 American Chemical Society.

On the other hand, when a hydrophobic side chain Leu is incorporated, in addition to the peptide bonds and the carboxylic group, the adsorption of hydrophobic leucyl residue on the hydrophobic parts of TiO_2_ surface would take place. As shown in Figure 14, for HT-TiO_2_, the adsorption of the Leu- containing peptides increased with the increase of the number of the peptide bond that is, Leu < Leu-Gly, Gly-Leu < Leu-Gly-Gly (Figure 14C).

**Figure 15:**
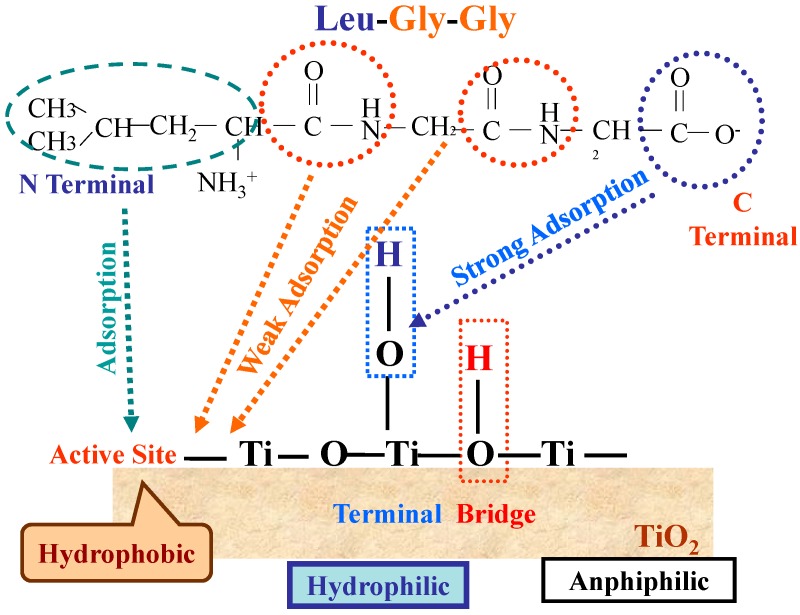
Schematic presentation of the plausible adsorption of the peptide (Leu-Gly-Gly) on the hydrophilic and hydrophobic surface of TiO_2_ (ST-01). Reprinted with permission from [43] © 2014 American Chemical Society.

However, the decomposition rates are almost the same (Figure 14D). These facts suggest that both the peptide bond and leucyl side chain could adsorb on the hydrophobic surface of TiO_2_ but photocatalytic decomposition should take place through the adsorption of the leucyl side chain which would adsorb preferably on the photocatalytic active part of the hydrophobic TiO_2_ surface. Thus leucyl residue would adsorb preferably on the active site of the hydrophobic part of TiO_2_ instead of the peptide bonds and photocatalysis proceeds. The adsorption feature of Leu-Gly-Gly, on TiO_2_ (ST-01) are illustrated in Figure 15.

However, as shown in Figure 14A,B it was found that Leu-Gly showed remarkably low adsorbability and decomposition rate as compared to Gly-Leu due to the specific conformation, in which the positively charged amino group and negatively charged carboxyl group interact strongly by electrostatic force [43]. Thus, when a peptide or proteins take a specific conformation, photocatalysis does not work effectively. For the effective use of TiO_2_ it would be necessary to acquire information on the surface conformation of the corresponding proteins/peptides to access the surface of the photocatalysts. By combining the information about the surface conformation of proteins/peptides and the active sites of TiO_2_ (hydrophobic or hydrophilic), we could design the TiO_2_ effective to diminish the specific virus, bacteria or environmental hazardous materials.

#### 3.2.3. Glutathione and Related Amino Acids

With increased applications of TiO_2_ nanoparticles, the concerns about their potential human toxicity and their environmental impact have also increased. Although details of human biological responses to TiO_2_ exposure are still unavailable, numerous *in vitro* examinations concerning cellular responses induced by TiO_2_ have been reported [44,45,46].

Glutathione is a tri-peptide capable of diminishing active oxygen species in living cells. In spite of the importance of glutathione in defense against oxidative stress, its actual affects and the mechanism for the TiO_2_-induced cytotoxicity and genotoxicity have not been completely elucidated yet.

**Figure 16:**
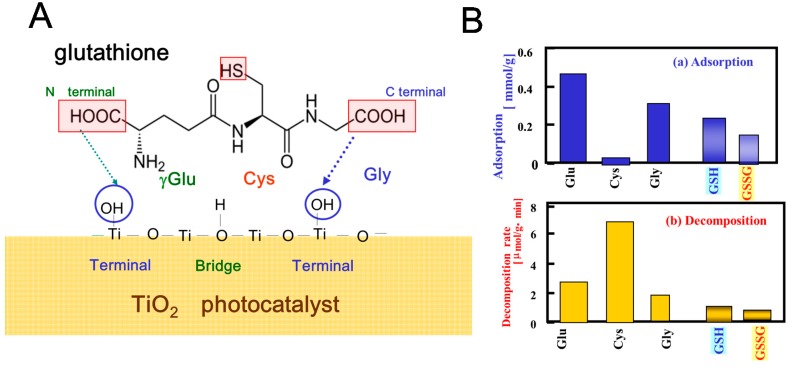
(**A**) Schematic presentation of the plausible adsorption of glutathione on the surface of TiO_2_ (ST-01); (**B**) (**a**) Adsorption and (**b**) decomposition rates of glutathione (GSH and GSSG) and the constituent amino acids (Glu, Cys, and Gly) in aqueous suspension of TiO_2_ (5 mg/0.4 mL D_2_O) under UV irradiation at 297 K. Reprinted with permission from [47] © 2012 American Chemical Society.

**Scheme 1:**
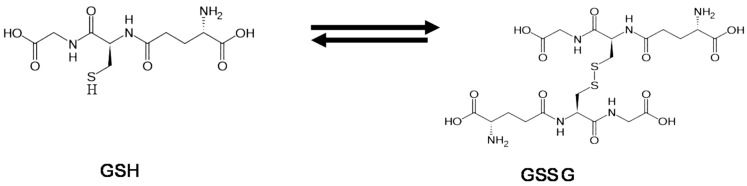
Glutathione cycle.

The photocatalytic decomposition of glutathione and related amino acids in TiO_2_ suspension was investigated with ^1^H NMR spectroscopy [47]. The results suggest, that as shown in Figure 16A, both glutathione in reduced (GSH) and oxidative forms (GSSG) are adsorbed on the TiO_2_ surface by carboxyl or amino groups but not by the thiol group (SH) of the side chain which plays a crucial role in the glutathione cycle (Scheme 1), to be degraded. This suggests that the function of glutathione cycle should be deteriorated in living cells by the adsorption. However, the decomposition rates are considerably slow as compared with those of the constituent amino acids (Glu, Cys and Gly) as shown in Figure 16B, possibly reflecting the self-defensive property against active oxygen species.

## 4. Conclusions

TiO_2_ photocatalysts have been utilized for the oxidation of organic pollutants. For the development of further practical applications, the improvement of the activity with the aid of an understanding of the detailed mechanism(s) of action is a prerequisite. The primary process of photocatalysis reported in the literatures still have some confusion. To clarify the reaction mechanism, the proper and reliable detection of primary active species, such as trapped electrons, trapped holes, •O_2_^−^ and •OH, in photocatalytic systems is required. By employing various spectroscopic techniques we have succeeded in elucidating some of the mechanisms of important photocatalytic reactions. Further investigations are proceeding in our laboratory.

## References

[1] Kaneko M., Ohkura I. (2002). Photocatalysis Science and Technology.

[2] Anpo M., Kamat P.V. (2010). Environmentally Benign Photocatalysts: Applications of Titanium Oxide-Based Materials.

[3] Pichat P. (2013). Photocatalysis and Water Purification: From Fundamentals to Recent Applications.

[4] Fujishima A., Zhang X., Tryk D.A. (2008). TiO_2_ Photocatalysis and related surface phenomena. Surf. Sci. Rep..

[5] Henderson M.A. (2011). A surface science perspective on TiO_2_ photocatalysis. Surf. Sci. Rep..

[6] Nosaka Y., Nosaka A.Y., Pichat P. (2013). Identification and roles of the active species generated on various photocatalysts. Photocatalysis and Water Purification.

[7] Nosaka Y., Yamashita Y., Fukuyama H. (1997). Application of chemiluminescent probe to monitoring superoxide radicals and hydrogen peroxide in TiO_2_ photocatalysis. J. Phys. Chem. B.

[8] Koizumi Y., Nosaka Y. (2013). Kinetics simulation of luminol chemiluminescence based on quantitative analysis of photons generated in electrochemical oxidation. J. Phys. Chem. A.

[9] Hirakawa T., Nosaka Y. (2008). Selective production of superoxide ions and hydrogen peroxide over nitrogen- and sulfur-doped TiO_2_ photocatalysts with visible light in aqueous suspension systems. J. Phys. Chem. C.

[10] Oguma J., Kakuma Y., Murayama S., Nosaka Y. (2013). Effects of silica coating on photocatalytic reactions of anatase titanium dioxide studied by quantitative detection of reactive oxygen species. Appl. Catal. B.

[11] Nosaka Y., Komori S., Yawata K., Hirakawa T., Nosaka A.Y. (2003). Photocatalytic •OH radical formation in TiO_2_ aqueous suspension studied by several detection methods. Phys. Chem. Chem. Phys..

[12] Louit G., Foley S., Cabillac J., Coffigny H., Taran F., Valleix A., Renault J.P., Pin S. (2005). The reaction of coumarin with the OH radical revisited: Hydroxylation product analysis determined by fluorescence and chromatography. Radiat. Phys. Chem..

[13] Gerald L.N., Jamie R.M. (2006). Fluorescence detection of hydroxyl radicals. Radiat. Phys. Chem..

[14] Matsushita M., Tran H., Nosaka A.Y., Nosaka Y. (2007). Photo-oxidation mechanism of L-alanine in TiO_2_ photocatalytic systems. Catal. Today.

[15] Nosaka A.Y., Nishino J., Fujiwara T., Yagi H., Akutsu H., Nosaka Y. (2006). Effects of thermal treatments on the recovery of adsorbed water and photocatalytic activities of TiO_2_ photocatalytic systems. J. Phys. Chem. B.

[16] Maldotti A., Molinari A., Amadelli R. (2002). Photocatalysis with organized systems for the oxofunctionalization of hydrocarbons by O_2_. Chem. Rev..

[17] Ikeda K., Sakai H., Ryo R., Hashimoto K., Fujishima A. (1997). Photocatalytic reactions involving radical chain reactions using microelectrodes. J. Phys. Chem. B.

[18] Zhang J., Nosaka Y. (2013). Quantitative detection of OH radicals for investigating the reaction mechanism of various visible-light TiO_2_ photocatalysts in aqueous suspension. J. Phys. Chem. C.

[19] Irie H., Miura S., Kamiya K., Hashimoto K. (2008). Efficient visible light-sensitive photocatalysts: Grafting Cu(II) ions onto TiO_2_ and WO_3_ photocatalysts. Chem. Phys. Lett..

[20] Yu H., Irie H., Shimodaira Y., Hosogi Y., Kuroda Y., Miyauchi M., Hashimoto K. (2010). An efficient visible-light-sensitive Fe(III)-grafted TiO_2_ photocatalyst. J. Phys. Chem. C.

[21] Macyk W., Kisch H. (2001). Photosensitization of crystalline and amorphous titanium dioxide by platinum(IV) chloride surface complexes. Chem. Eur. J..

[22] Ishibai Y., Sato J., Nishikawa T., Miyagishi S. (2008). Synthesis of visible-light active TiO_2_ photocatalyst with Pt-modification: Role of TiO_2_ substrate for high photocatalytic activity. Appl. Catal. B.

[23] Nishikawa M., Hiura S., Mitani Y., Nosaka Y. (2012). Photocatalytic reaction mechanism of Fe(III)-grafted TiO_2_ studied by means of ESR spectroscopy and chemiluminescence photometry. J. Phys. Chem. C.

[24] Nosaka Y., Takahashi S., Sakamoto H., Nosaka A.Y. (2011). Reaction mechanism of Cu(II)-grafted visible-light responsive TiO_2_ and WO_3_ photocatalysts studied by means of ESR spectroscopy and chemiluminescence photometry. J. Phys. Chem. C.

[25] Nishikawa M., Takanami R., Nakagoshi F., Suizu H., Nagai H., Nosaka Y. (2014). Dominated factors for high performance of Fe^3+^ grafted metal doped TiO_2_ based photocatalyst. Appl. Catal. B.

[26] Niishiro R., Konta R., Kato H., Chun W.J., Asakura K., Kudo A. (2007). Photocatalytic O_2_ evolution of rhodium and antimony-codoped rutile-type TiO_2_ under visible light irradiation. J. Phys. Chem. C.

[27] Oropeza F.E., Egdell R.G. (2011). Control of valence states in Rh-doped TiO_2_ by Sb co-doping: A study by high resolution X-ray photoemission spectroscopy. Chem. Phys. Lett..

[28] Liu M., Qiu X., Miyauchi M., Hashimoto K. (2013). Energy-level matching of Fe(III) ions grafted at surface and doped in bulk for efficient visible-light photocatalysts. J. Am. Chem. Soc..

[29] Burgeth G., Kisch H. (2002). Photocatalytic and photoelectrochemical properties of titania-chloroplatinate(IV). Coord. Chem. Rev..

[30] Nishikawa M., Sakamoto H., Nosaka Y. (2012). Reinvestigation of photocatalytic reaction mechanism for Pt-complex-modified TiO_2_ under visible-light irradiation by means of ESR Spectroscopy and chemiluminescence photometry. J. Phys. Chem. A.

[31] Bard A.J., Parsons R., Jordan J. (1985). Standard Potentials in Aqueous Solution.

[32] Fujishima A., Honda K. (1972). Electrochemical photolysis of water at a semiconductor electrode. Nature.

[33] Nakabayashi Y., Nosaka Y. (2013). OH radical formation at distinct faces of rutile TiO_2_ crystal in the procedure of photoelectrochemical water oxidation. J. Phys. Chem. C.

[34] Imanishi A., Fukui K. (2014). Atomic-scale surface local structure of TiO_2_ and its influence on the water photooxidation process. J. Phys. Chem. Lett..

[35] Ohno T., Sarukawa K., Matsumura M. (2001). photocatalytic activities of pure rutile particles isolated from TiO_2_ powder by dissolving the anatase component in HF Solution.. J. Phys. Chem. B.

[36] Zhang J., Nosaka Y. (2014). Mechanism of the OH radical generation in photocatalysis with TiO_2_ of different crystalline types. J. Phys. Chem. C.

[37] Hirakawa T., Yawata K., Nosaka Y. (2007). Photocatalytic reactivity for O_2_^−^ and OH radical formation in anatase and rutile TiO_2_ suspension as the effect of H_2_O_2_ addition. Appl. Catal. A.

[38] Dadjour M.F., Ogino C., Matsumura S., Nakamura S., Shimizu N. (2006). Disinfection of legionella pneumophila by ultrasonic treatment with TiO_2_. Water Res..

[39] Ishiguro H., Nakano R., Yao Y., Kajioka A., Fujishima A., Sunada K., Minoshima M., Hashimoto K., Kubota Y. (2011). Inactivation of Qβ bacteriophage by photocatalysis using TiO_2_ thin film under weak with long wavelength UV irradiation. Photochem. Photobiol. Sci..

[40] Diebold U. (2003). The surface science of titanium dioxide. Surf. Sci. Rep..

[41] Mastikhin V.M., Mudrakovsky I.L., Nosov A.V. (1991). ^1^H-NMR magic angle spinning (MAS) studies of heterogeneous catalysis. Prog. NMR Spectrosc..

[42] Köppen S., Bronkalla O., Langel W. (2008). Molecular simulation of protein-surface interactions. J. Phys. Chem. C.

[43] Nosaka A.Y., Tanaka G., Nosaka Y. (2014). Study by use of ^1^H-NMR spectroscopy of the adsorption and decompoaition of glycine, leucine, and derivatives in TiO_2_ photocatalysis. J. Phys. Chem. B.

[44] Fenoglio I., Greco G., Livraghi S., Fubini B. (2009). Non-UV-induced radical reactions at the surface of TiO_2_ nanoparticles that may trigger toxic responses. Chem. Eur. J..

[45] Petković J., Žegura B., Filipič M. (2011). Influence of TiO_2_ nanoparticles on cellular antioxidant defense and its involvement in genotoxicity in HepG2 cells. J. Phys. Conf. Ser..

[46] Horie M., Kato H., Fujita K., Endoh S., Iwahashi H. (2012). *In vitro* evaluation of cellular response induced by manufactured nanoparticles. Chem. Res. Toxicol..

[47] Nosaka A.Y., Tanaka G., Nosaka Y. (2012). The behaviors of glutathione and related Amino Acids in TiO_2_ photocatalytic system. J. Phys. Chem. B.

